# Sub-Saharan red cell antigen phenotypes and glucose-6-phosphate dehydrogenase deficiency variants in French Guiana

**DOI:** 10.1186/s12936-016-1365-8

**Published:** 2016-06-07

**Authors:** Florence Petit, Pascal Bailly, Jacques Chiaroni, Stéphane Mazières

**Affiliations:** CNRS, IRD, Avignon Université, IMBE UMR 7263, Aix Marseille Université, 13397 Marseille, France; CNRS, EFS, ADES UMR 7268, Aix Marseille Université, 13916 Marseille, France; Etablissement Français du Sang Alpes Méditerranée, 13392 Marseille, France

**Keywords:** Red cell blood group systems, Glucose-6-phosphate dehydrogenase deficiency, French Guiana, Noir Marron community, *Plasmodium vivax*, Primaquine

## Abstract

**Background:**

The treatment of *Plasmodium vivax* infections requires the use of primaquine, which can lead to severe haemolysis in glucose-6-phosphate dehydrogenase (G6PD)-deficient individuals. However, most of the Latin American countries, which are still endemic for *vivax* malaria, lack information on the distribution of G6PD deficiency (G6PDd). No survey has been performed so far in French Guiana. Herein, 80 individuals of the French Guianan Noir Marron population were scrutinized for red cell surface antigens of six blood group systems (ABO, Rh, Kell, Kidd, Duffy and MNS) and G6PD genetic polymorphisms. First, the sub-Saharan origin of the red cell phenotypes was assessed in relation with the literature. Then, given that the main sub-Saharan G6PDd variants are expected to be encountered, only the *G6PD* sequences of exons 4, 5, 6 and 9 were screened. This work aims at appraising the *G6PD* gene variation in this population, and thus, contributing to the G6PD piecemeal information in Latin America.

**Results:**

Ninety-seven percent (97 %) of the red cells are Fy(a− b−), either D+ C− E− c+ e+ or D+ C+ E− c+ e+ and 44 % exhibited the Fya−/Jkb−/S− combined phenotype. Noteworthy is the detection of the G6PD(Val68Met) variant characterized by c.202G > A transition, G6PD(Asn126Asp) variant characterized by c.376A>G transition and G6PD(Asp181Val) variant characterized by c.542A>T transversion of the *G6PD* gene in 22.5 % of the sample, characteristic of the A^−(202)^, A and Santamaria G6PDd variants, respectively.

**Conclusions:**

French Guianan Noir Marron population represents a pool of Rh-D antigen positive, Duffy-negative and G6PD-deficient erythrocytes, the latter accounting for one in every eight persons. The present study provides the first community-based estimation of the frequency of G6PDd polymorphisms in French Guiana. These results contribute to the G6PD genetic background information puzzle in Latin America.

## Background

Glucose-6-phosphate dehydrogenase deficiency (G6PDd), an X-linked, hereditary and recessive genetic trait, is the most common genetic enzymopathy in humans. Glucose-6-phosphate dehydrogenase (G6PD) is an enzyme catalyzing the first reaction in the pentose phosphate pathway, providing reducing power to all cells in the form of NADPH (nicotinamide adenine dinucleotide phosphate). In red cells, defense against oxidative damage relies only on NADPH generated by G6PD activity [1]. More than 200 variants and 186 substitutions in the *G6PD* gene have been described [2, 3]. Though mostly asymptomatic, G6PDd may cause red cell membrane damage, haemoglobin crystals, haemolytic anaemia, neonatal jaundice, or haemoglobinuria in cases of induced oxidative stress from nutrition and drugs. Importantly, primaquine is a major anti-malarial treatment. Individuals who have inherited the G6PDd phenotype can exhibit sensitivity to primaquine, which leads to symptoms that range from moderate to lethal depending on the deficiency class [4, 5], and as such are designated primaquine-sensitive. Clinical relapse due the release of liver hypnozoites is the major characteristic of *Plasmodium vivax* and amino-8-quinoline-based primaquine and tafenoquine remain the only effective drugs against this parasitic form to date [1, 6]. The concordance of the geographical distribution of G6PDd variants with lower levels of parasitaemia and reduced risk of infection led to the hypothesis that this enzymatic feature is selectively advantageous in past and present malarial endemic areas [7–10]. Though the exact mechanisms remain elusive, G6PDd would favour early phagocytosis of parasitized red blood cells at a stage where the parasite has not yet multiplied [11].

Recently, reviews mapped the state-of-the-art of the G6PD variation evidenced at the enzymatic and molecular levels [12–14]. G6PDd seems restricted to Africa, Southern Europe, Asia, and Pacific islands and virtually absent in the Native American populations of the Americas [12, 15]. In Latin America, though drug-induced haemolysis represents most of the acute anaemia, only minimal data is available to accurately address the G6PD geographical distribution among non-urban populations [13]. Most Latin American countries are endemic areas for malarial parasites and transmission vectors [16]. Even if Mexico, Haiti, and Costa Rica would have eliminated malaria, other countries are still in control phase and require the use of primaquine knowing the risk of haemolysis that may result in G6PD-deficient persons. In Latin America but French Guiana, primaquine is prescribed for every patient infected with *P. vivax* without looking for G6PDd or ethnicity assignment [17]. In French Guiana, *P. vivax* has become the dominant malaria species with more than 60 % of attacks with a substantial increase of cases since mid-2001, mostly observed in children [18], Amerindian populations [19], gold panners, French armed forces [20], Hmong [21], and to a lesser extent, in the Noir Marron community [22]. Most of the malaria transmission occurs along the two frontier rivers. Along the middle Maroni River—western border with Suriname—*Plasmodium falciparum* malaria incidence remains higher than *P. vivax*, particularly in the Noir Marron territory. In contrast, *P. vivax* is more frequent in the upper reaches of the Maroni River where it infests the Wayana and Emerillon Amerindian populations [22]. *Plasmodium vivax* is also preponderant along the eastern border with Brazil, Oyapock River, and in the eastern inland areas. Though part of former anthropological studies [23–25], G6PD genetic variation in French Guiana in relation with malaria risk has never been carried out so far [22]. This lack of information prevents implementation of efficient programmes for the control or elimination of *P. vivax* malaria in Latin America [13].

The present study aims to partially tackle this issue with original data from a well-defined population from French Guiana assumed to carry sub-Saharan genetic polymorphisms. For this aim, the Noir Marron community which has arisen from the merger of slaves who escaped from Dutch plantations [26], was investigated. First, validation of the sub-Saharan origin of the red cells was allowed by the frequencies of antigen phenotypes of six membrane genetic systems linked with a sub-Saharan ancestry. At the same time, knowledge of these main red cell antigen frequencies would be crucial for blood product requirements and minimum stock levels to ensure blood transfusion compatible with recipient blood characteristics. Then the main sub-Saharan G6PDd variants indexed in [2] and assumed to be encountered were screened by molecular biology.

## Methods

### Sample collection

Study protocol, sample collection and de-identification were approved by the Comité de Protection des Personnes (C.P.P.) Sud-Ouest et Outre-Mer I, file no 1-11-39, ID-RCB no 2011-A00996-35. After approval of the informed consent, venous blood samples were collected in EDTA tubes from 80 Aluku, Ndjuka, Saramaka and Paramaka living in Papaichton, Loka, Boniville and Maripasoula, along the Maroni River. Along with sampling, data relative to sex, age, state of health, place of residence and population memberships have been recorded.

### Surface antigen typing

Red cell antigens of the ABO, Rh, Kell, Kidd, Duffy, and MNS blood group systems were investigated. Due to the presence of natural ABO antibodies and strong immunogenicity of the Rh-D antigen, the ABO–D phenotype was typed first, followed by a second level of identification of the Rh–Kell antigenic profiles, ending with extended Duffy, Kidd and Ss (MNS) phenotypes.

Typing was carried out with Diagast (Loos, France) reagents anti-A (Clone 9113D10), -B (Clone 9621A8), -AB (Clones 9113D10 + 152D12), -D (Clones P3 × 61 + P3 × 21223B10 + P3 × 290 + P3 × 35), -Fya, -S, and -s; Eurobio (France) reagents anti-C (Clones MS273), -c (Clone MS35), -E (Clones MS12 + MS260), -e (MS62 + MS69) and -K (Clone AEK4), and Bio-Rad (Marnes-la-Coquette, France) reagents anti-Fyb, -Jka, and -Jkb.

### DNA extraction

Genomic DNA was extracted from 200 µl of whole blood using a Blood DNA mini kit (QIAamp, Qiagen, Courtaboeuf, France) according to the manufacturer’s instructions.

### Primer design

In order to confirm the sub-Saharan origin of the samples, the red cell antigen phenotypes were confronted with the frequencies encountered in sub-Saharan populations [12, 15]. Previous surveys revealed Central and West Africa as the putative origins of the Noir Marron, as well as no admixture in this population [26, 27]. This assumption allowed the expectation in the samples of the main sub-Saharan G6PDd variants that would have been brought during Atlantic Slave Trade [2]. Hence, *G6PD* exons 4, 5, 6 and 9 coding for the main sub-Saharan G6PDd variants were sequenced. Protocol is also appropriate for main variants encountered in South East Asia [28], the geographical origin of another French Guianan population with plausible G6PDd, the Hmong [29]. Primers are listed in Table 1 and were designed according to works of [30, 31], the Primer3 [32] and OligoPerfect™ Designer (Life Technologies, Carlsbad, CA, USA) tools.

**Table 1:** Primer sequences used in the present study

*G6PD* exon	Forward	Reverse	References	Amplicon size (bp)
4	*5′-CTGCCCGCACTGGTTACA-3′*	5′-AGGAGAGGAGGAGAGCATCC-3′	[2]	259
5	*5′-CTGTCTGTGTGTCTGTCTGTC-3′*	5′-GAGGGCAACGGCAAGCCTT-3′	[2]	272
6	*5′-GTCTGAATGATGCAGCTGTGA-3′*	5′-CCAGGTGAGGCTCCTGAGTA-3′	[30], Present study	296
9	5′-TCTCCCTTGGCTTTCTCTCA-3′	*5′-GTGCGTGAGTGTCTCAGTGG-3′*	Present study	295

Primers in italic were used in the sequence reaction

### PCR amplification

Exons were independently amplified from 50 ng of genomic DNA in a reaction mix containing 1 X buffer, 1.5 M of magnesium ions, 0.05 mM of each dNTP, 0.16 μM of each primer and 1 unity (U) of Taq polymerase (Invitrogen™, Cergy Pontoise, France). Amplifications were carried out in a 96-Well Veriti^®^ (Applied Biosystem^®^, Courtaboeuf, France) or DNAEngine Peltier thermal cyclers (Bio-RAD, Marnes-la-Coquette, France). Temperature profile was 96 °C for 5 min (min.), followed by 30 cycles of 96 °C for 1 min., 60 °C for 1 min., 72 °C for 1 min., and a final extension step of 7 min. at 72 °C. PCR products were controlled through electrophoresis on 2 % agarose gels stained with ethidium bromide.

### PCR purification and sequence reaction

Five µl of PCR products were purified in an enzymatic mix of 1 U of Thermosensitive Alkaline Phosphatase combined with 10 U of Exonuclease 1 (Euromedex). Purification steps were 15 min. at 37 °C ending with 15 min. at 85 °C in a Veriti™ 96-Well thermal cycler (Applied Biosystems).

After purification, forward fragments of exons 4, 5 and 6, and reverse strand of exon 9 were sequenced using primers listed in Table 1 and the BigDye™ terminator Cycle Sequencing Ready Reaction v1.1 (Applied Biosystem^®^, Courtaboeuf, France) following manufacturer’s protocol. After a Sephadex™ gel filtration, strands were segregated by capillary electrophoresis in a ABI PRISM 3130 genetic analyzer (Applied Biosystem^®^, Courtaboeuf, France) using POP-4^®^ polymer and 36-cm length capillary.

### Sequence alignment and allele estimation

Sequences were aligned to the *G6PD* reference sequence (accession number X55448.1) using CodonCode Aligner 3.5.3 (CodonCode Corporation, www.codoncode.com) and BioEdit 7.2.5 [33] following caution highlighted in [34] for exons 4 and 5. In order to assess heteroplasmy, the signature of heterozygous individuals, both forward and reverse DNA strand was sequenced and electrophoregrams proofread. *G6PD* allele frequencies were calculated by direct counting.

### Ethics approval and consent to participate

Study protocol, sample collection and de-identification were approved by the Comité de Protection des Personnes (C.P.P.) Sud-Ouest et Outre-Mer I, file no 1-11-39, ID-RCB no 2011-A00996-35. The veinous blood samples were collected after approval of the informed consent of the 80 individuals Aluku, Ndjuka, Saramaka and Paramaka.

## Results

Table 2 presents the occurrence of red cell phenotypes. Fifty-nine samples were O type and all were D+ but one. The most frequent Rh phenotype was D+ C− E− c+ e+ (49 cases out of 80) followed by D+ C+ E− c+ e+ (19 cases). The only D− phenotype was D− C− E− c+ e+. All samples carried the Cellano phenotype (K− k+) of the Kell system, 90 % the Jk^a^ antigen and all samples but one (98.8 %) were positive for the s antigen. As far as Duffy is concerned, 98.1 % were Fy(a− b−) and three Duffy positive individuals were found. Taken together, the Fya−/Jkb−/S− red cells account for 44 % of the sample.

**Table 2:** Phenotype distribution for six red cell blood group systems in the sample under study

System	Phenotype	N
ABO	A	6
	AB	4
	B	10
	O	59
Rh	D+ C+ E− c+ e+	19
	D+ C+ E+ c+ e+	2
	D+ C− E+ c+ e−	1
	D+ C− E+ c+ e+	7
	D+ C− E− c− e+	1
	D+ C− E− c+ e+	49
	D− C− E− c+ e+	1
Kell	K− k+	80
	K+ k−	0
Kidd	Jk(a+ b−)	46
	Jk(a+ b+)	26
	Jk(a− b+)	8
Duffy	Fy(a+ b−)	2
	Fy(a− b+)	1
	Fy(a− b−)	77
(MNS) Ss	S+ s−	1
	S+ s+	10
	S− s+	69

Table 3 presents the observed *G6PD* genotypes and Table 4 the inferred *G6PD* allele frequencies. Non-mutated sequences represent 77.5 % of the sample studied. Variants account for 22.5 % of the chromosomes sequenced. The most frequent G6PDd variants are A and A^−(202)^ (10.9 %). One case of Santamaria (G6PD(A376G) transition, G6PD(Asp181Val) variant characterized by c.542A>T transversion) has been detected. Full enzymatic-deficient individuals which include males and homozygous females, represent 12.5 % of our sample. Neither the G6PD(Ser188Phe) Mediterranean variant characterized by c.563C>T transition nor the G6PD(Leu323Pro) Betica variant characterized by c.968T>C transition was detected. None of the three Duffy-positive samples were carrier of a G6PDd variant.

**Table 3:** Number of *G6PD* genotypes in the Noir Marron sampled for this study

	Male	Female	
Genotype	Hemizygous	Heterozygous	Homozygous
B	15	–	–
B/B	–	–	38
A/B	–	4	–
A	6	–	–
A/A	–	–	2
A^−(202)^	1	–	–
A^−(202)^/B	–	11	–
A^−(202)^/A	–	1	–
A^−(202)^/A^−(202)^	–	–	1
Santamaria/B		1

**Table 4:** *G6PD* allele frequency in the sample under study

cDNA nucleotide substitution	Variant name	Class	Allele frequency
–	B		0.775
c. 376A>G	A	IV	0.109
c. 202G>A, c. 376A>G	A^−(202)^	III	0.109
c. 376A>G, c. 542A>T	Santamaria	II	0.007

## Discussion

The worldwide geographical distribution of membrane, haemoglobin and enzymatic red cell phenotypes is closely associated with *P. falciparum* and *P. vivax* [6, 8, 9, 12, 22, 35, 36]. In the New World, Latin America is endemic to *P. vivax* and *P. falciparum* and seven mosquito vector species have been identified [16, 37]. While all Native American populations may lack G6PDd, the other Latin American populations may possess *G6PD* mutations due to their non-Amerindian ancestry. This is especially the case of the Afro-descendant populations of the Amazonian basin whose sub-Saharan ancestry could reach 100 % [38–40].

Appraisal of parasitic loads, vector density and genetic background of the host is crucial to measure the level of transmission and the proportion of primaquine-sensitive individuals in malarial endemic areas. Although anti-malarial cure is unsafe taken with G6PDd, workers have recently pointed out the paucity of detailed information in Latin America [13]. In addition, no survey has been carried out so far in French Guiana [22], a level-4 G6PDd risk area [41]. In order to supply with G6PD information in Latin America, herein is presented the first community-based estimation of the frequency of G6PDd polymorphims in French Guiana.

Notable is the prevalence of the D+ C− E− c+ e+, Fy(a− b−), and the combined Fya−/Jkb−/S− (44 %) phenotypes. These are usually encountered in western sub-Saharan populations where frequencies reach 30 % for Fya−/Jkb−/S−, 70 % for D+ C− E− c+ e+, and up to 100 % for Fy(a− b−) [42, 43]. The present survey also revealed occurrence of G6PD A and A^−(202)^ variants and one case of Santamaria; a variant fortuitously found in this study given its frequency worldwide [44–46]. Founder effect, the mechanism by which a population rose from a small number of founders and thus of alleles, would have thus concentrated sub-Saharan red cell genetic components including membrane antigen and *G6PD* genetic polymorphisms in the Suriname and Guianese Noir Marron [26].

Fy(a− b−) individuals are putatively insensitive to *P. vivax* infestation, so unconcerned by primaquine treatment. Nevertheless, several cases of Fy(a− b−) individuals infested by *P. vivax* have been reported in Malagasy, Africa, and more importantly in Brazil [47–50], making Duffy-negative individuals the novel panel of individuals suitable for primaquine treatment. Hence, estimation of G6PDd may be extended to a priori unconcerned populations such as the ones encountered in Latin America with West African genetic ancestry.

Capture of the human red cell genetic variation provides crucial information for transfusion safety. Transfusion risks are firstly embodied by the ABO-Rh-Kell phenotype variation, further by Duffy, Kidd and Ss phenotype depending on the patient, pathology and risk of alloimmunization. The present sample showed high values of O type, Rh-D antigen positive and Duffy-negative cases (alternatively said, positive for anti-FY antibodies). In addition, it has been reported that G6PDd reduces the efficacy of exchange transfusion in neonates and children recipients [51] with hyperkalaemia from the haemolysis of transfused blood cells and hyperbilirubinaemia due to the inefficient discard of the bilirubin excess by the immature liver, leading in one rare case to dark urine and acute intravascular haemolysis [52]. In patients with sickle cell disease who need repeated transfusions, donor blood G6PDd may also induce haemolysis [51]. Hence, studies recommended a routine screening for G6PDd for these at-risk patients [52], which may be discussed in countries where the prevalence of this deficiency is high like in Mediterranean basin and African ancestry populations. Describing the polymorphism of G6PDd may help to choose which populations have to be screened and what kind of polymorphism have to be detected. To date, in the context where French Guianans could represent a panel of G6PD-deficient donors, the small incidence of the Santamaria variant is not of primary importance given that blood donation is blocked by viral seroprevalence [53–56].

Enzymatic variation could usually be identified by non-molecular methods [57, 58]. Herein, the main sub-Saharan G6PD variants in a population from French Guiana were investigated through the direct screening of four *G6PD* exon sequences. French Guiana is also inhabited by western European populations in urban centres and a SouthEast asian community—the Hmong—in Cacao and Javouhey [29] for which level of G6PDd remains unknown. Besides exons 4 and 5, the PCR also targeted exons 6 and 9 where the Mediterranean, Mahidol, Rehevot and Viangchan substitutions occur [28]. Thus, the protocol performed may be practicable to the other communities of the area.

## Conclusions

Due to the scarcity of the community-based studies led so far, screening of level and nature of G6PDd is a main challenge in Latin America. The present study aimed to fill the blank with original data from a sub-Saharan population living on the border of Suriname and French Guiana. The sub-Saharan origin of the studied red cells was confirmed from six blood group systems. Three *G6PD* polymorphisms (G6PD(Val68Met) variant characterized by c.202G>A transition, G6PD(Asn126Asp) variant characterized by c.376A>G transition, and G6PD(Asp181Val) variant characterized by c.542A>T transversion) defining three G6PDd variants (A^−(202)^, A and Santamaria) were detected in 22.5 % of the sample. The present survey pointed out that French Guianan Noir Marrons represent a reservoir of Rh-D antigen positive, Duffy-negative and primaquine-sensitive phenotypes, the latter accounting for one in every eight persons.

## Abbreviations

G6PD: glucose-6-phosphate dehydrogenase
G6PDd: glucose-6-phosphate dehydrogenase deficiency
NADPH: nicotinamide adenine dinucleotide phosphate
